# Postoperative Serotonin Syndrome Triggered by Propofol in a Patient on Chronic Serotonergic Medications and Bupropion: A Case Report

**DOI:** 10.7759/cureus.83151

**Published:** 2025-04-28

**Authors:** Stephen G Chong, Brannon L Inman, Rachel E Bridwell

**Affiliations:** 1 Emergency Medicine, Madigan Army Medical Center, Joint Base Lewis McChord, USA; 2 Emergency Medicine, Brooke Army Medical Center, Fort Sam Houston, USA; 3 Emergency Medicine, Uniformed Services University of the Health Sciences, Bethesda, USA

**Keywords:** bupropion, cyproheptadine, fluoxetine, postoperative, propofol, serotonin syndrome, serotonin toxicity

## Abstract

Serotonin syndrome is a potentially life-threatening condition that is commonly associated with the use of serotonergic agonistic medications, such as selective serotonin reuptake inhibitors (SSRI), serotonin-norepinephrine reuptake inhibitors (SNRI), and opioids. In contrast, propofol, a commonly used perioperative induction and intravenous sedation medication, is not frequently reported to cause serotonin syndrome. We present a rare case in a 34-year-old female who developed serotonin syndrome in the postoperative setting after the administration of propofol for induction and maintenance of anesthesia. The patient presented postoperatively with diaphoresis and non-fatigable ocular and extremity clonus, at which time she received benzodiazepines and cyproheptadine after discontinuing the offending agent. The patient successfully recovered from the condition following the administration of these medications.

## Introduction

Serotonin syndrome (SS) is a well-known, life-threatening diagnosis typically associated with serotonergic medications. In cases of SS, the risk of development appears to increase with the addition of multiple serotonin agonists (SA), with a disproportionally increased risk of SS as more SAs are administered [1]. Selective serotonin reuptake inhibitors (SSRI) and serotonin-norepinephrine reuptake inhibitors (SNRI) are more commonly implicated; However, other medications such as opioids (fentanyl, meperidine, methadone, and tramadol) have also been reported to precipitate SS [1-3]. SS is a heterogeneous condition with varying degrees of severity and presentation, with resultant uncertainty regarding the true number of cases. While the number of cases of SS is unknown, recent years have borne witness to increased use and availability of SAs, raising concern about the increased burden of SS [3]. Propofol is a commonly used sedative agent that is not classically thought to be associated with serotonergic properties [1-3]. While SS is commonly associated with medications like SSRIs and SNRIs, its occurrence following propofol administration is extremely rare, especially when compounded by the use of multiple serotonergic drugs. Cases of SS following propofol are thought to be rare, likely owing to a lack of historical association and sparse representation in the literature. We present a case of SS in a 34-year-old female postoperatively, who developed SS after propofol administration while on chronic serotonergic medications. 

## Case presentation

A 34-year-old female with a history of asthma and anxiety managed as an outpatient with fluoxetine 40 mg daily and bupropion 150 mg daily presented to the Emergency Department (ED) for progressive headache with progressively worsening right eye visual acuity due to transverse dural sinus stenosis. Following admission to the hospital, the patient underwent neurosurgical stent placement for right transverse sinus stenosis with a high-pressure gradient. During the perioperative period, the patient was induced with 200 mg of propofol intravenously. Anesthesia was maintained using total intravenous anesthesia (TIVA) using a propofol drip titrated to a bispectral index monitor (BIS) value of 40-60, with no complications during anesthetization or procedure. The patient had no documented administration of opioids or other SAs intraoperatively. During the postoperative recovery period, she was found to have seizure-like activity. She was given a weight-based bolus of 1 mg/kg propofol and transferred to the Neuro Critical Care Unit (NCCU).

Upon presentation to the NCCU, initial vitals included a temperature of 99.9°F (37.7°F), tachycardia of 118 beats per minute, blood pressure of 117/60 mmHg, respiratory rate of 21 breaths per minute, and oxygen saturation of 100%. A physical exam demonstrated an intubated, sedated, diaphoretic female with brisk bilateral 3 mm pupils and evidence of ocular clonus. An exam of her extremities showed hyperreflexia, increased tone, and sustained non-fatigable spontaneous clonus of the upper and lower extremities.

Due to the presence of spontaneous and inducible clonus with autonomic dysregulation, there was raised concern for SS. The patient was administered serial aliquots of lorazepam for a total of 6 mg and was loaded with 12 mg cyproheptadine. A subsequent electroencephalogram (EEG) demonstrated no seizure activity. The propofol infusion was discontinued, and the patient’s physical exam improved with normalization of vital signs and resolution of both ocular and extremity clonus. The patient progressively became more alert and could move all extremities without difficulty. Following discontinuation of propofol, the patient was transitioned to a dexmedetomidine infusion and extubated within 24 hours of propofol cessation, on postoperative day 1.

## Discussion

We report a rare presentation of SS secondary to acute propofol administration with chronically prescribed multiple SAs. Several SAs have a well-known association with SS, including SSRIs and SNRIs, which are more commonly prescribed today for mood disorders [1-3]. However, other drug classes with various mechanisms of action can contribute to SS, including increased 5-HT synthesis, inhibition of 5-HT metabolism, activation of 5-HT1 receptors, antagonism of 5-HT2A receptors, and inhibition of 5-HT reuptake [1].

The diagnosis of SS poses a challenge as the presentation can vary widely. Classically, patients can present with a spectrum of clinical findings to include mild symptoms, such as akathisia or tremors, to autonomic hyperactivity, hyperreflexia, clonus, altered mental status, and muscular hypertonicity in the setting of multiple SAs [2]. In attempts to capture patients presenting with SS with increased sensitivity and specificity, diagnostic criteria such as Hunter’s Serotonin Toxicity Criteria may be utilized (Table 1) [4].

**Table 1:** Hunter's Serotonin Toxicity Criteria

HUNTER CRITERIA
Presence of Serotonergic Agent AND meets one of the following:
Spontaneous clonus
Tremor + Hyperreflexia
Ocular clonus + (Agitation OR Diaphoresis)
Inducible clonus + (Agitation OR Diaphoresis)
Hypertonia + temperature >38°C + (Ocular Clonus OR Inducible Clonus)

The mainstay of treatment for SS is benzodiazepines and supportive care [1-3]. Cyproheptadine, an oral or enterally administered 5-HT2 and H1 receptor antagonist, may be recommended for its anti-serotonergic properties in the treatment of SS [1]. In the management of SS, the use of cyproheptadine has been a subject of controversy. Given the potential side effects, interactions with other medications (i.e. antihistamines and anticholinergics), and logistical issues with enteral administration, it is crucial to seek the advice of a toxicologist before prescribing cyproheptadine. As such, toxicologists recommend its administration only on a case-by-case basis.

There have been a few case reports associated with SS secondary to propofol administration: two occurred intraoperatively and the other postoperatively [5-7]. The offending agents that contributed to the presentation of SS in this patient are likely due to propofol in combination with her home medications of fluoxetine and bupropion. Bupropion is a norepinephrine and dopamine uptake inhibitor, and it is not commonly known as a SA. Still, there have been cases of SS occurring with SSRIs as well as bupropion in isolation that resolved with cyproheptadine administration [8,9]. Bupropion has been known to be an inhibitor of the cytochrome P450 D6 pathway that could increase levels of SSRIs [8]. The patient's home medication combination of bupropion and fluoxetine alone was unlikely to cause SS with consistent medication compliance without prior signs or symptoms. However, it is possible that the home medication combination could have elevated the levels of fluoxetine, leading to an interaction with the addition of propofol. 

In this case, there were no complications or hemodynamic changes that indicated SS intraoperatively. However, the postoperative ocular clonus, rigidity, and spontaneous myoclonic jerks in combination with home SA use and propofol met the criteria for diagnosis of SS based on Hunter’s Serotonin Toxicity Criteria [3,4]. With the recent addition of propofol as a possible offending SA, the patient had fulfilled several Hunter Criteria including spontaneous clonus and ocular/inducible clonus with diaphoresis. While the current medical literature does not firmly establish the serotonergic properties of propofol, the medication may modulate serotonin release or receptor activity, thus becoming an offending agent for SS in the setting of multiple serotonergic medications. With very few cases of propofol-associated SS, this relationship could be a new polypharmacy combination to precipitate this condition.

## Conclusions

SS is a rare, life-threatening condition characterized by autonomic instability and spontaneous or inducible clonus following the administration of serotonergic medications. Prompt recognition, discontinuation of the offending agents, administration of benzodiazepines, and toxicology consultation for potential cyproheptadine use are essential for effective management. Although SS due to propofol is exceedingly rare, this case demonstrates that it should be considered, particularly in patients receiving multiple serotonergic medications. This case highlights the importance of maintaining a high index of suspicion for SS in patients on chronic serotonergic therapy, even after routine anesthetic procedures, and underscores the need for timely diagnosis and intervention.
